# Measurement properties of the German version of the IKDC subjective knee form (IKDC-SKF)

**DOI:** 10.1186/s41687-018-0058-1

**Published:** 2018-07-13

**Authors:** Danica Kümmel, Stefan Preiss, Laurent P. Harder, Michael Leunig, Franco M. Impellizzeri

**Affiliations:** 1 Department of Teaching, Research and Development, Schulthess Clinic, Lengghalde 2, 8008 Zurich, Switzerland; 2 Musculoskeletal Centre, Orthopaedics Lower Extremities, Schulthess Clinic, Lengghalde 2, 8008 Zurich, Switzerland

**Keywords:** IKDC, PROM, Validity, Reliability, Responsiveness

## Abstract

**Purpose:**

To examine the measurement properties of the German International Knee Documentation Committee Subjective Knee Form (IKDC-SKF) in knee disorder patients.

**Methods:**

Three hundred twelve consecutive patients undergoing surgery for anterior cruciate ligament, meniscus and/or cartilage injuries completed the IKDC-SKF, Lysholm Score, Tegner Activity Scale, and Short Form-12 Health Survey before and 6 months post-surgery. IKDC-SKF measurement properties were calculated and patients were also asked to rate the relevance/comprehensibility of the questionnaire items.

**Results:**

Reliability was good with high Cronbach’s alpha and intraclass correlation coefficients, and standard error of measurement values of 4.4 to 6.0. The smallest detectable change (SDC) ranged from 12.3 to 16.7 points. Validity was good with 90% of all hypotheses confirmed. Confirmatory factor analysis did not show adequate fitting indices within the model. Over half of the items were rated as essential, and all were well comprehended. The majority of hypotheses for responsiveness were confirmed. No floor and ceiling effects were observed. The area under the curve ranged from 0.82 to 0.89 and the minimal important difference was smaller than the SDC.

**Conclusions:**

The German IKDC-SKF is a reliable outcome measure with good hypotheses testing and responsiveness, but its MIC and structural/content validity need further analysis.

## Introduction

The International Knee Documentation Committee Subjective Knee Form (IKDC-SKF) assesses symptoms, daily function and sports activity in patients with knee disorders [13, 14]. The tool has good measurement properties [9] and is freely available in several languages including German [10, 16, 19, 22]. Unlike for most of the language versions available, the translation process of the German IKDC-SKF is unknown and the instrument has not been validated. There is a lack of validated German knee-specific tools to assess and compare multiple knee conditions and treatments. Within the scope of our clinical studies, a valid German IKDC-SKF would serve as a comprehensive assessment of patients for prospective use in our clinic.

We examined the measurement properties of the German IKDC-SKF in anterior cruciate ligament (ACL), meniscus and/or cartilage injury patients undergoing surgery according to COnsensus-based Standards for the selection of health status Measurement INstruments (COSMIN) checklist [18].

## Materials and methods

### Patients and data collection

Seven hundred seven consecutive patients underwent ACL, meniscal and/or articular cartilage surgery in our clinic between September 2015 and December 2016. All patients completed a set of questionnaires at home within four weeks before (i.e. baseline) and 6 months after surgery. Data were excluded if items were incomplete or patients did not agree to provide written consent, had insufficient knowledge of the German language, did not reside in Switzerland, were under 16 years or suffered from any condition that hindered study participation. All consecutive patients who returned the questionnaire set within 12 days after completion received the IKDC-SKF again. For reliability testing, patients who completed the second IKDC-SKF questionnaire within 14 days after the first occasion were included. Psychometrics were calculated for the patient subgroups: ACL surgery only (ACL); meniscal surgery only (Meniscus); articular cartilage surgery only (Cartilage); and a combination of the three surgery types (mixed surgery subgroup = Mix).

Because the German IKDC-SKF cross-cultural adaptation is not published, we evaluated comprehensibility and relevance of each questionnaire item at baseline, 6 or 12 months post-surgery with two additional patient groups. Patients from these groups underwent surgery between April 2016 and April 2017 and were selected in further sampling procedures to increase the participation rate.

### Questionnaires

IKDC-SKF examines 19 items on 5-point Likert (items 1, 4, 5, 7, 8 & 9a-i), 0–10 rating (items 2, 3, 10a & 10b) or dichotomous scales (item 6). The overall score is based on 18 items (item 10a is not included) and ranges from 0 to 100 points with higher points corresponding to less symptoms, better function and a higher level of sports activity [13].

The Short Form-12 (SF-12) Health Survey measures generic health status with 12 questions that are combined, scored and weighted to produce physical and mental component summary scale (PCS-12 and MCS-12) scores (0 to 100). A higher score indicates better health-related quality of life [24]. The SF-12 showed acceptable criterion validity, structural validity and reliability in ill and healthy subjects [3, 24].

The Lysholm Score contains eight items measuring knee function (i.e. limping, locking, pain, stair climbing, need for support, instability, swelling and squatting) [2, 3, 15]. The total score ranges from 0 (poor) to 100 (excellent outcome without symptoms or disability). This score showed acceptable reliability, validity and responsiveness in patients with ACL/meniscal injuries and chondral disorders [2, 3, 15, 25].

The single-item Tegner Activity Scale measures the highest activity level achieved during work/sport activities [2, 3]; 0 indicates a sick leave patient and 10 indicates participation in elite-level competitive sports. This tool showed acceptable measurement properties in patients with ACL/meniscal injuries [2, 3, 26]. Cross-culturally adapted and validated German versions of all questionnaires were used [7, 25, 26].

At 6 months, patients were asked to rate their global treatment outcome (GTO) by answering: *“How much did the operation help your knee problem?”* on a 5-point Likert scale with the following options of (1) helped a lot; (2) helped; (3) helped only little; (4) did not help; or (5) made things worse [12].

For IKDC-SKF item comprehensibility, patients were asked, *“In your opinion, how comprehensible is the question formulated?”* and could answer with: (1) totally comprehensible, (2) mostly comprehensible, (3) moderately comprehensible, (4) slightly comprehensible, or (5) not at all comprehensible. In a similar manner, patients were asked to rate the relevance of each IKDC-SKF item, i.e. *“In your opinion, how essential is this item in order to describe your situation?”* [1] and to specify missing items, i.e. *“In your opinion, which questions/items are also very important, but are missing in this questionnaire?”.*

We excluded incomplete questionnaire sets when more than two items were missing from the IKDC-SKF and any single item was missing from the other questionnaires.

### IKDC-SKF measurement properties

Internal consistency was calculated using Cronbach’s alpha with values between 0.7 and 0.95 indicating appropriate internal consistency [21]. Intraclass correlation coefficients (ICC) were calculated using a single measurement, absolute agreement, 2-way mixed-effects model to assess test-retest reliability; an ICC of at least 0.7 was considered appropriate [21]. Agreement was assessed using the Standard Error of Measurement (SEMagreement). The smallest detectable change (SDC) was calculated using the formula: SDC = 1.96 * √2 * SEM [5].

Item relevance was measured by counting “ne”, i.e. number of patients rating an item as “essential” and comparing this value to “Ncritical” (minimum number of patients required to agree an item as “essential”) [1]; Ncritical was 9 for 10 patients per subgroup and 26 for 40 patients in total. Comprehensibility was calculated by the relative frequency of patient ratings.

Structural validity was assessed by confirmatory factor analysis (CFA) using the maximum likelihood method with Satorra-Bentler adjustment. Validity was demonstrated if data fitted the recursive one factor structure proposed in a number of studies [9, 13, 20, 22] with or without item 6 as part of the model. CFA was also performed without item 6 in order to achieve a better model fit, since item 6 is dichotomous and had low factor loadings in previously performed analyses [13, 20, 22]. Validity was good if at least 75% of the tested hypotheses were confirmed [21]. Strong correlations overall and for each subgroup (≥ 0.6) were expected between the IKDC-SKF and SF-12 (PCS-12) as well as IKDC-SKF and Lysholm Score [4, 13, 16, 23]. Weak to moderate correlations (0.1–0.49) were expected between the IKDC-SKF and Tegner Activity Scale [3, 6]. Furthermore, we expected correlations of 0.1–0.29 for the IKDC-SKF and SF-12 (MCS-12) [13, 16].

Responsiveness was assessed with predefined hypotheses on the change scores between baseline and 6 months. Moderate correlations (≥ 0.4) for the entire population and each subgroup were expected between the change scores of the IKDC-SKF and SF-12 (PCS-12), and IKDC-SKF and Lysholm Score [22, 23]. A strong, inverse correlation (≤ − 0.6) was expected between the change scores of the IKDC-SKF and GTO.

In general, the effect size (ES) and standardised response mean (SRM) were expected to be large (≥ 0.8) [4, 14, 15].

Floor and ceiling effects were considered absent if percentages were below 15% [21]. To assess minimal important change (MIC), receiver operating characteristics (ROC) were analysed with the GTO as the anchor question. Patients who stated that the operation helped or helped a lot were considered to have a good outcome; all other responses indicated a poor outcome. The MIC was estimated as the optimal ROC cut-off point represented by the smallest value equivalent to the sum of 1-sensitivity and 1-specificity [5]. Area under the curve (AUC) was calculated as a measure of discriminant ability.

All analyses were performed using Stata Corp. 2015 Stata Statistical Software: Release 14 (StataCorp LP, College Station, TX, USA).

## Results

**Fig. 1:**
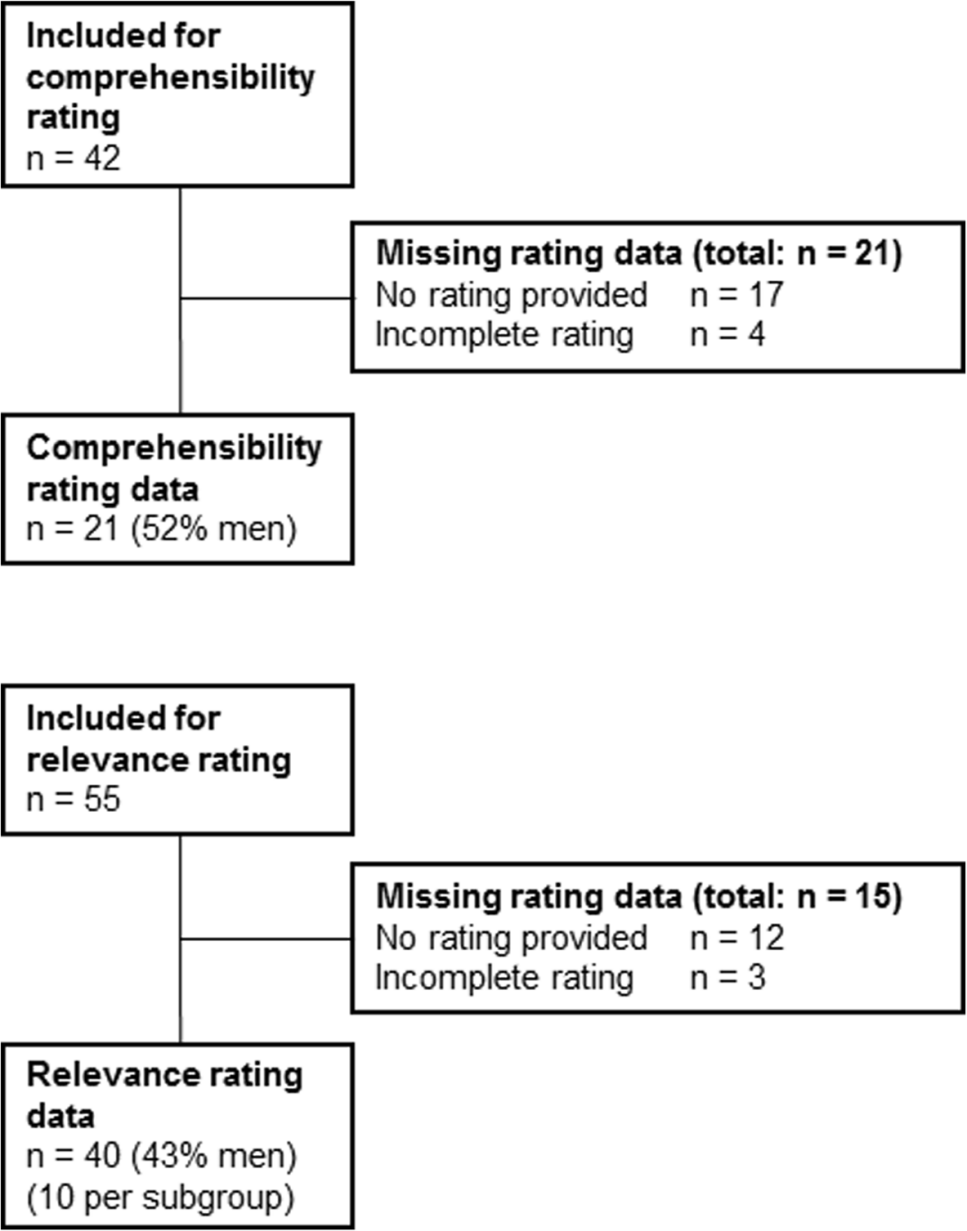
Flow chart of the patient subgroup assessment of German IKDC-SKF item comprehensibility and relevance

**Fig. 2:**
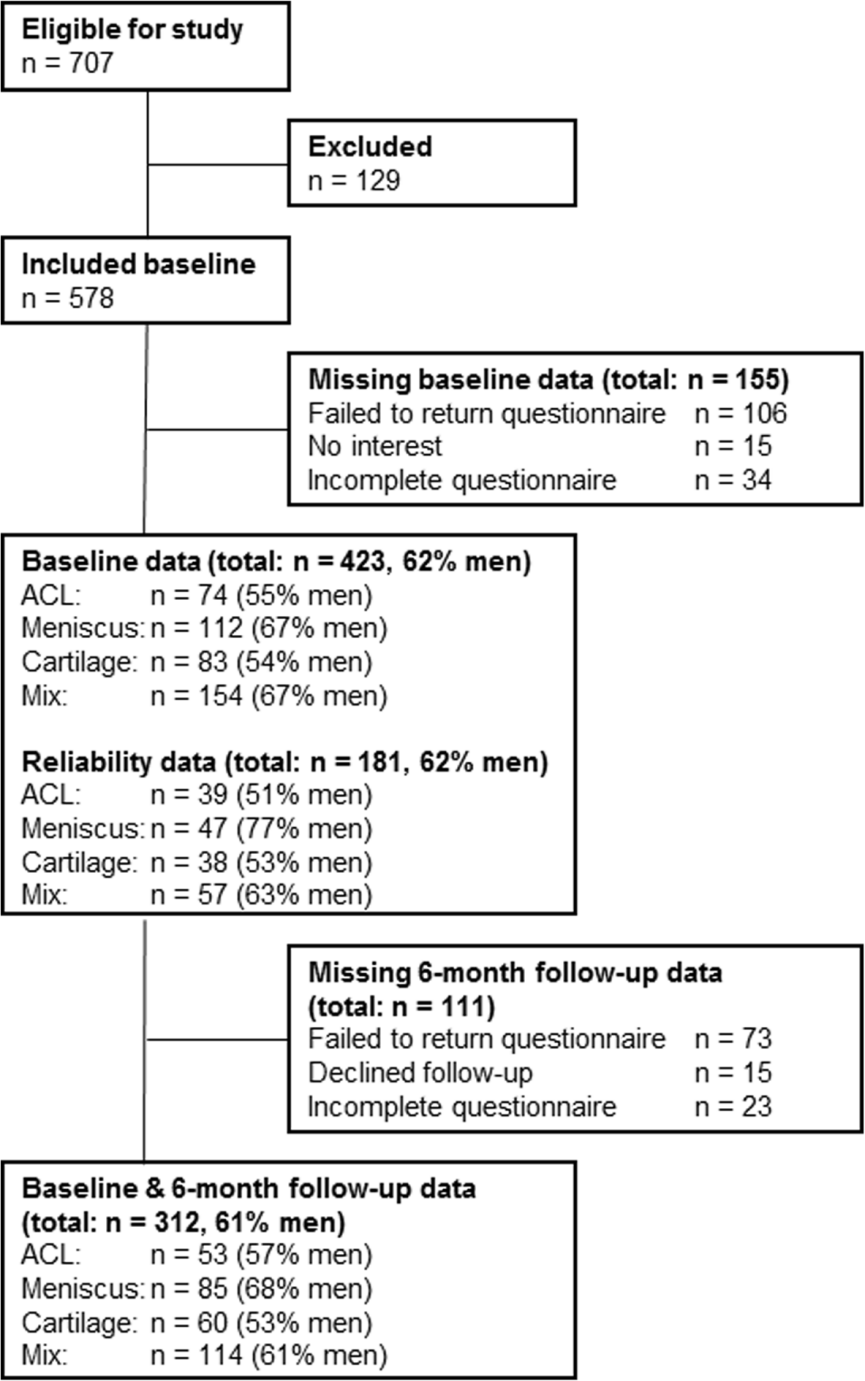
Selection algorithm showing patient eligibility and the available sample sizes for assessing the measurement properties of the German IKDC-SKF

The number of patients for assessing content validity and all measurement properties are shown in Figs. 1 and 2, respectively. The mean age of the patient population was 38 years (SD 14 years; range 16–74 years). Mean IKDC-SKF scores at baseline and 6 months were 53.5 and 71.3, respectively (Table 1).

**Table 1:** Questionnaire scores for the total population as well as each patient subgroup

	Examination time point		Reliability		Comprehensibility	Relevance
	Baseline	6 months	Test (T1)	Retest (T2)		
IKDC-SKF, mean (SD)						
Total	53.5 (15.7)	71.3 (16.7)	55.7 (17.0)	56.1 (16.5)	63.3 (19.5)	63.6 (17.5)
ACL	60.2 (13.5)	74.2 (11.5)	63.3 (14.1)	63.8 (13.2)		67.0 (20.8)
Meniscus	52.2 (16.6)	76.7 (16.8)	55.7 (18.6)	55.0 (17.4)		65.4 (12.4)
Cartilage	46.8 (14.4)	60.6 (19.5)	46.8 (14.5)	47.8 (14.1)		49.9 (18.0)
Mix	54.7 (15.3)	71.5 (14.7)	56.6 (16.4)	57.2 (17.1)		72.1 (11.0)
Lysholm Score, mean (SD)						
Total	61.6 (17.9)	80.5 (16.4)				
ACL	67.7 (15.2)	82.5 (14.2)				
Meniscus	62.0 (18.3)	84.0 (15.6)				
Cartilage	53.7 (16.7)	69.8 (18.6)				
Mix	62.5 (18.0)	82.8 (14.6)				
Tegner Activity Scale, mean (SD)						
Total	3.2 (2.0)	3.8 (1.4)				
ACL	3.6 (2.0)	3.9 (1.5)				
Meniscus	3.0 (1.8)	4.0 (1.5)				
Cartilage	2.9 (1.6)	3.2 (1.5)				
Mix	3.4 (2.3)	3.8 (1.2)				
PCS-12, mean (SD)						
Total	41.8 (10.1)	45.2 (6.6)				
ACL	44.0 (9.6)	45.0 (6.2)				
Meniscus	40.7 (10.2)	46.6 (5.9)				
Cartilage	40.3 (9.0)	41.9 (7.7)				
Mix	42.5 (10.7)	46.0 (6.2)				
MCS-12, mean (SD)						
Total	51.5 (10.3)	55.1 (7.7)				
ACL	50.3 (9.8)	56.0 (7.0)				
Meniscus	53.0 (9.8)	55.1 (8.2)				
Cartilage	50.4 (11.3)	54.4 (8.4)				
Mix	51.6 (10.3)	55.0 (7.4)				

*IKDC-SKF* = International Knee Documentation Committee Subjective Knee Form, *PCS-12* = 12-item short-form health survey (SF-12) physical component summary scale*, MCS-12* = 12-item short-form health survey (SF-12) mental component summary scale, *SD* = standard deviation, *Total* = all eligible study patients, *ACL* = anterior cruciate ligament surgery patients only, *Meniscus* = meniscal surgery patients only, *Cartilage* = articular cartilage surgery only, *Mix* = patients from a combination of the three surgery types (mixed surgery subgroup)

For the entire patient cohort, Cronbach’s alpha was 0.87; similar values were calculated for the various subgroups (0.84–0.89).

ICC was 0.94 (95%CI, 0.91–0.95) for the total cohort and similar for ACL, Meniscus, Cartilage and Mix subgroups. SEMagreement values were also similar among the patient groups (range 4.4–6.0 points) as were the SDC values (range 12.3–16.7 points) (Table 2).

**Table 2:** IKDC-SKF reliability and minimal important change

	IKDC-SKF				
	Total	ACL	Meniscus	Cartilage	Mix
Cronbach’s Alpha^a^ (95% CI)	0.87 (0.85; 0.89)	0.84 (0.76; 0.92)	0.89 (0.86; 0.92)	0.87 (0.83; 0.91)	0.87 (0.84; 0.90)
ICC^b^ (95% CI)	0.94 (0.91; 0.95)	0.93 (0.86; 0.96)	0.93 (0.89; 0.96)	0.92 (0.85; 0.96)	0.93 (0.88; 0.96)
SEM^b^	5.4	4.4	5.6	6.0	5.5
SDC^b^	14.9	12.3	15.6	16.7	15.3
MIC^c^	6.7	−1.3	11.3	12.5	12.5

*ICC* = intraclass correlation coefficient, *CI* = confidence interval, *SEM* = standard error of measurement, *SDC* = smallest detectable change, *MIC* = minimal important change, *IKDC-SKF* = International Knee Documentation Committee Subjective Knee Form, *Total* = all eligible study patients, *ACL* = anterior cruciate ligament surgery patients only, *Meniscus* = meniscal surgery patients only, *Cartilage* = articular cartilage surgery only, *Mix* = patients from a combination of the three surgery types (mixed surgery subgroup)

^a^Sample sizes for Cronbach’s alpha calculations are equivalent to baseline data “n” in Fig. 2

^b^Sample sizes for ICC, SEM and SDC calculations are equivalent to reliability data “n” in Fig. 2

^c^Sample sizes for the MIC calculation are equivalent to baseline & 6-month follow-up data “n” in Fig. 2

IKDC-SKF items were rated as totally or mostly comprehensible by the majority (≥ 86%) of patients. Some items were also rated as slightly or not comprehensible by 4.8% (item 1, 3, 7, 9d, 9i, 10a) and 15.3% (item 2) of patients. In the total patient sample, 52.6% of items were rated “essential” by a sufficient number of patients (ne ≥ 26). With a ne value ≥9, the following percentage of items were rated as essential by the ACL, Cartilage and Mix subgroups: 5.3% (item 7); 36.8% (items 1, 2, 5, 8, 9i, 10a & b); and 5.3% (items 9d & h), respectively; none of the items were essential for the Meniscus group. Eight patients suggested additional items covering the following topics: ACL: pain quality; Meniscus: avoided sport activities; Cartilage: previous surgery, inability to work; employment Mix: possible impulsive movements, strength training, previous knee surgery.

The lowest factor loading for item 6 was 0.15, and over 0.4 for all other items. Satorra-Bentler adjusted goodness of fit parameters were inadequate when taking item 6 into consideration (χ^2^ (df) (chi-square statistic [degrees of freedom]): 1101.84 (135), p: 0.00; Comparative Fit Index (CFI): 0.696; Tucker-Lewis index (TLI): 0.655; root mean squared error of approximation (RMSEA): 0.131; standardised root mean squared residual (SRMR): 0.096; coefficient of determination (CD): 0.918) or not (χ^2^ (df): 1086.74 (119), p: 0.00; CFI: 0.696; TLI:0.652; RMSEA: 0.139; SRMR 0.1; CD 0.918). The majority of hypotheses evaluating construct validity (90%) (Table 3) and responsiveness (76%) were confirmed (Table 4).

**Table 3:** Hypothesis testing for evaluating validity of the IKDC-SKF for the total population as well as each patient subgroup

	IKDC-SKF				
	Total	ACL	Meniscus	Cartilage	Mix
Lysholm	**0.80** (≥ 0.6)	**0.75** (≥ 0.6)	**0.79** (≥ 0.6)	**0.81** (≥ 0.6)	**0.8** (≥ 0.6)
Tegner	**0.34** (0.1–0.49)	**0.39** (0.1–0.49)	**0.37** (0.1–0.49)	**0.42** (0.1–0.49)	**0.25** (0.1–0.49)
PCS-12	**0.70** (≥ 0.6)	**0.71** (≥ 0.6)	**0.67**(≥ 0.6)	**0.68** (≥ 0.6)	**0.72** (≥ 0.6)
MCS-12	**0.17** (0.1–0.29)	**0.13** (0.1–0.29)	**0.23** (0.1–0.29)	0.30 (0.1–0.29)	0.09 (0.1–0.29)

Correlation estimates in parentheses indicate a priori formulated hypotheses

Correlation estimates in **bold** indicate confirmed hypotheses

*IKDC-SKF* = International Knee Documentation Committee Subjective Knee Form, *Lysholm* = Lysholm Score, *Tegner* = Tegner Activity Scale, *PCS-12* = 12-item short-form health survey (SF-12) physical component summary scale, *MCS-12* = 12-item short-form health survey (SF-12) mental component summary scale, *Total* = all eligible study patients, *ACL* = anterior cruciate ligament surgery patients only, *Meniscus* = meniscal surgery patients only, *Cartilage* = articular cartilage surgery only, *Mix* = patients from a combination of the three surgery types (mixed surgery subgroup)

**Table 4:** Responsiveness of the IKDC-SKF for the total population as well as each patient subgroup

	IKDC-SKF				
	Δ total	Δ ACL	Δ Meniscus	Δ Cartilage	Δ Mix
Δ Lysholm	**0.74** (≥ 0.4)	**0.69** (≥ 0.4)	**0.79** (≥ 0.4)	**0.75** (≥ 0.4)	**0.72** (≥ 0.4)
Δ PCS-12	**0.60** (≥ 0.4)	**0.57** (≥ 0.4)	**0.54** (≥ 0.4)	**0.68** (≥ 0.4)	**0.59** (≥ 0.4)
GTO	−0.55 (≤ − 0.6)	−0.45 (≤ − 0.6)	−0.56 (≤ − 0.6)	**−0.66** (≤ − 0.6)	−0.51 (≤ − 0.6)
ES	**1.04** (≥ 0.8)	**1.11** (≥ 0.8)	**1.39** (≥ 0.8)	0.75 (≥ 0.8)	**1.06** (≥ 0.8)
SRM	**0.96** (≥ 0.8)	**0.84** (≥ 0.8)	**1.23** (≥ 0.8)	0.71 (≥ 0.8)	**0.99** (≥ 0.8)

Correlation estimates in parentheses indicate a priori formulated hypotheses

Correlation estimates in **bold** indicate confirmed hypotheses

Δ = change score

*IKDC-SKF* = International Knee Documentation Committee Subjective Knee Form, *PCS-12* = 12-item short-form health survey (SF-12) physical component summary scale, *GTO* = global treatment outcome, *ES* = effect size, *SRM* = standardised response mean, *Total* = all eligible study patients, *ACL* = anterior cruciate ligament surgery patients only, *Meniscus* = meniscal surgery patients only, *Cartilage* = articular cartilage surgery only, *Mix* = patients from a combination of the three surgery types (mixed surgery subgroup)

No floor and ceiling effects were observed. AUC values ranging from 0.82 to 0.89 for all groups were considered appropriate, but calculated MICs varied considerably (total [6.8], ACL [− 1.3], Meniscus [11.3], Cartilage [12.5], and Mix [6.7]).

## Discussion

Our study showed good reliability and hypotheses testing of the IKDC-SKF, which is in line with previous investigations [4, 9, 10, 13, 15–17, 19, 22, 23].

CFA revealed similar factor loadings compared to other studies [13, 20, 22]. Nevertheless, our data do not fit the hypothesised model based on the fit indices. The application of uni- and two-dimensional scales for the IKDC-SKF is debatable [9, 11, 13, 20, 22]. This indicates that the resulting lack of structural validity can be attributed to the construct rather than the translation.

Over half of the items were rated as essential by at least 65% of patients in the total population, and up to almost 40% of the items were rated as essential by a minimum of 90% of patients in the subgroups.

The variation between subgroups may indicate different priorities, various main symptoms and that shorter questionnaires are preferred; the additional items suggested by our patients seem to support the differences in their concerns and symptoms. All items were rated as comprehensible, and we believe the general wording is sufficient. We consider content validity to be moderate. A comparison to previous studies is difficult, since evaluation of patient opinion on item relevance has been addressed in few studies including ACL patients and different analytical methods, and item comprehensiveness is lacking [22, 23].

The German IKDC-SKF showed good responsiveness and no floor and ceiling effects; these characteristics are confirmed in previous studies [8, 9, 14, 22, 23]. MIC was smaller than SDC for all test groups, which indicates a suboptimal ability to distinguish clinically relevant changes from measurement error at the individual level. However, this finding is in line with previous studies comparing SDC with MIC at 4 and 6 months post-surgery [8, 22].

A potential limitation could be our GTO, which was applied as the anchor for MIC calculation and refers to a change based on the surgery alone. Since all patients received standard clinical treatment, we believe this GTO can be used to satisfactorily assess change.

## Conclusion

The German IKDC-SKF is a reliable tool showing good hypotheses testing and responsiveness for patients with ACL, meniscus and/or cartilage disorders undergoing surgery. However, structural/content validity and MIC require further analysis.
